# Vestibular Evoked Myogenic Potential on Ocular, Cervical, and Soleus Muscles to Assess the Extent of Neurological Impairment in HTLV-1 Infection

**DOI:** 10.3389/fneur.2020.00433

**Published:** 2020-05-21

**Authors:** Tatiana Rocha Silva, Marco Aurélio Rocha Santos, Luciana Macedo de Resende, Ludimila Labanca, Júlia Fonseca de Morais Caporali, Rafael Teixeira Scoralick Dias, Denise Utsch Gonçalves

**Affiliations:** ^1^Graduate Program in Infectious Diseases and Tropical Medicine, School of Medicine, Universidade Federal de Minas Gerais, Belo Horizonte, Brazil; ^2^Graduate Program in Phonoaudiological Sciences, School of Medicine, Universidade Federal de Minas Gerais, Belo Horizonte, Brazil

**Keywords:** vestibular function tests, motor evoked potentials, human T-lymphotropic virus 1, postural balance, vestibular nerve, saccule and utricle

## Abstract

**Introduction:** Vestibular Evoked Myogenic Potential (VEMP) can be used to test central vestibular pathways from the midbrain to the lumbar spine, according to the muscle tested.

**Purpose:** to compare the spinal cord alteration in individuals with HTLV-1-associated myelopathy (HAM) and with HTLV-1-asymptomatic infection using the VEMP recorded from different muscles.

**Methods:** VEMP was recorded in 90 individuals of whom 30 had HAM, 30 were HTLV-1 asymptomatic carriers, and 30 negative controls. VEMP was recorded in the oculomotor muscle (oVEMP), testing the vestibulo-ocular reflex, and in the cervical muscle (cVEMP) and soleus muscle (sVEMP), testing the vestibulospinal reflex, respectively, in the cervical and in the lumbar spinal level. The type of stimulation was auditory for oVEMP and cVEMP, and galvanic for sVEMP. The compared variables were the latencies of the electrophysiological waves.

**Results:** HTLV-1-asymptomatic group was similar to the controls regarding oVEMP (*p* = 0.461), but different regarding cVEMP (*p* < 0.001) and sVEMP (*p* < 0.001). HAM group has presented the worst latencies and was different from the HTLV-1-asymptomatic group in the VEMP of all the tested muscles (*p* < 0.001). The concomitant occurrence of VEMP alterations in the three recorded muscles of the same individual was found in 2 (6.7%) asymptomatic carriers and in 20 (66.7%) patients with HAM (*p* = 0.001). The analysis of VEMP alteration per group and per muscle has showed that, in HTLV-1-asymptomatic group, oVEMP was altered in 3 (10.0%) individuals, cVEMP in 10 (33.3%) and sVEMP in 13 (43.3%). In HAM group, oVEMP was altered in 23 (76.6%) individuals, cVEMP in 27 (90%), and sVEMP in 30 (100%).

**Conclusion:** HTLV-1-neurological damage has followed an ascendant progression beginning at the lumbar spine in the stage of a clinically asymptomatic infection, whereas HAM has affected not only the spine, but also the midbrain.

## Introduction

Human lymphotropic T-cell virus type 1 (HTLV-1) is widely disseminated worldwide, and it is estimated that 15 to 20 million people have been infected (1). The means through which the interaction between the virus and the host develops is a determining factor in the state of the asymptomatic carrier or disease (2, 3).

Numerous diseases are correlated with HTLV-1 infection: uveitis, Sjogren's syndrome, infectious dermatitis, polymyositis, arthropathies, thyroiditis, polyneuropathies, lymphocytic alveolitis, cutaneous T-cell lymphoma, strongyloidiasis, scabies, leprosy, tuberculosis, and HTLV-1 associated myelopathy (HAM) (4–6).

In HAM, the site of major involvement is the lower thoracic spine, although the entire neuro-axis can also be involved (7). Alterations in the cervical spine have been identified even in the asymptomatic phase (8). Moreover, the parenchymal lesions may not be limited to the spinal cord (9–11). In fact, there is evidence of diffuse involvement of the central nervous system (CNS) caused by HTLV-1 infection (7, 12). Reports of cognitive impairment have been associated with this infection, including changes in fluid intelligence, semantic memory, attention, and information processing (13, 14).

Postural instability is a frequent clinical manifestation in HAM (15). The complaint of dizziness can be one of the first clinical manifestations of neurological alteration, indicating a possible evolution from asymptomatic carrier to HAM (15). Some patients considered to be “asymptomatic carriers” present electrophysiological changes in the vestibulospinal tract, which participates in the postural control (15).

Vestibular Evoked Myogenic Potential (VEMP) is an electrophysiological test of a tri-synaptic reflex that evaluates the peripheral vestibular system and the central function related to the labyrinth connections. It is considered a test that evaluates the brainstem response (16, 17). The muscles that are the most commonly used to record VEMP are the oculomotor, also called ocular VEMP (oVEMP); the sternocleidomastoid, also called cervical VEMP (cVEMP); and the soleus muscle, also called soleus VEMP (sVEMP) (18–20).

In oVEMP, the activation of the vestibulo-ocular reflex is presumed to follow the vestibular primary afferent, possibly medial longitudinal fasciculus, nucleus, and oculomotor nerves, including the mesencephalic connections (18). In cVEMP, the vestibulocollic reflex goes through the primary vestibular afferent, medial vestibulospinal tract and spinal accessory nerve (18). In sVEMP, the vestibulospinal reflex is conducted through the inferior vestibular nerve, lateral vestibular nucleus, lateral vestibulospinal tract, and reticulospinal tract (21–23). Thus, VEMP varies according to the type of stimulation and to the muscle used to record the electromyographic (EMG) response.

To better characterize the neurological disease associated with HTLV-1 infection, this study aims at comparing VEMP recorded from different muscles in patients with HAM and in HTLV-1 asymptomatic carriers, assessing the CNS at different levels.

## Methods

### Ethical Aspects

This research was conducted in accordance with the principles expressed in the Declaration of Helsinki and was approved by the Research Ethics Committee from Universidade Federal de Minas Gerais (COEP UFMG), logged under protocol number CAAE 92928518.3.0000.5149. This protocol number refers to a main project that includes subprojects of which the present study is one of them. All participants provided voluntary written consent and declared that they were aware of the study procedures and their choice to participate.

### Study Design

This study was a comparative cross-sectional analysis. The oVEMP, the cVEMP, and the sVEMP were compared among individuals classified as definite HAM, HTLV-1-asymptomatic carriers, and healthy seronegative controls (24).

### Sample Size

The sample size was calculated using G^*^ Power software 3.1.9.2 (Heinrich-Heine Universitat Düsseldorf, Düsseldorf, Germany, 2007) to achieve a power of 80% and a significance level of 5% based on the mean and standard deviation of the P13-N23 waves in the cVEMP response of patients with HAM and healthy controls (15). The final calculation estimated an inclusion of 30 participants per group.

### Participants

The studied groups were recruited from a cohort of former blood donors infected with HTLV-1 who have been followed by the Interdisciplinary HTLV Research Group (GIPH) since 1997, in Belo Horizonte, Brazil. The GIPH evaluates the natural history, clinical manifestations, and epidemiological aspects of HTLV infection (25).

Ninety participants of the GIPH cohort were invited to participate in this study. They consisted of 30 individuals with definite HAM, 30 with HTLV-1-asymptomatic infection, and a control group of 30 individuals not infected by HTLV-1 (24). The control group consisted of active and healthy blood donors. They were submitted to clinical interviews and neurological examinations before being submitted to VEMP.

The classification of the participants infected by HTLV-1 regarding neurological impairment followed the Expanded Disability Status Scale (EDSS) (24, 26) and the OMDS scale (24, 27): asymptomatic individual (EDSS and OMDS - 0 on both scales) and definite diagnosis of HAM (EDSS and OMDS greater than 1 on both scales).

Individuals with a positive serology for the Human Immunodeficiency Virus (HIV), HTLV-2, or any other blood-tested disease were excluded, as well as an undetermined serology for HTLV-1 and a positive Venereal Disease Research Laboratory test. To avoid confusion factors related to the exam, we excluded individuals using metal prosthesis; with neurological diseases, neoplasms, otitis, and tympanic membrane perforation; with a history of cranio-encephalic trauma or otologic surgery, and peripheral vestibular disease; as well as individuals unable to perform cervical rotation and that were unable to remain in an orthostatic position.

### Vestibular Evoked Myogenic Potential (VEMP)

VEMP can be evoked by either auditory or electrical stimulus (galvanic) (20, 21). The technique to perform the test using auditory stimulus is simpler when compared to the galvanic test. However, the recording of VEMP in lower limbs (soleus or gastrocnemius muscle) triggered by auditory stimulus is more difficult because of the lower accumulated energy up to the final neurological path when compared to the galvanic stimulus, which is a more robust one. Thus, the galvanic stimulus is more appropriate to record VEMP in the lower limbs (21). However, it is more uncomfortable for the patient when compared to the auditory stimulus. Because of this, in the present study, we have used the auditory stimulus to obtain oVEMP and cVEMP, and the galvanic stimulus to obtain sVEMP. The parameters considered in the comparison of VEMP analyses were the latency and the reproducibility of the EMG wave.

### Ocular VEMP (oVEMP) and Cervical VEMP (cVEMP)

The oVEMP and cVEMP were performed simultaneously (model Labat/Epic Plus, Labat Asia Pvt Ltd., Mohali, India), using two channels. The stimuli were presented through ER 3A insertion phones (of brand Etymotic Research Inc.), with disposable foam ear tips. Tone burst stimuli at an intensity of 120 decibels, a normalized hearing level (dB nHL), were used. In this study, a bandpass filter of 10 to 1,500 Hertz (Hz) was used. To obtain each record, 100 stimuli were presented at a frequency of 500 Hz at a rate of four stimuli per second. The scan window was 50 milliseconds (ms). Each subject underwent at least two stimulations per side in order to verify the replication of the potential. The impedance values, which had to be below 5 kiloohms, were checked before each recording (16).

**Figure 1:**
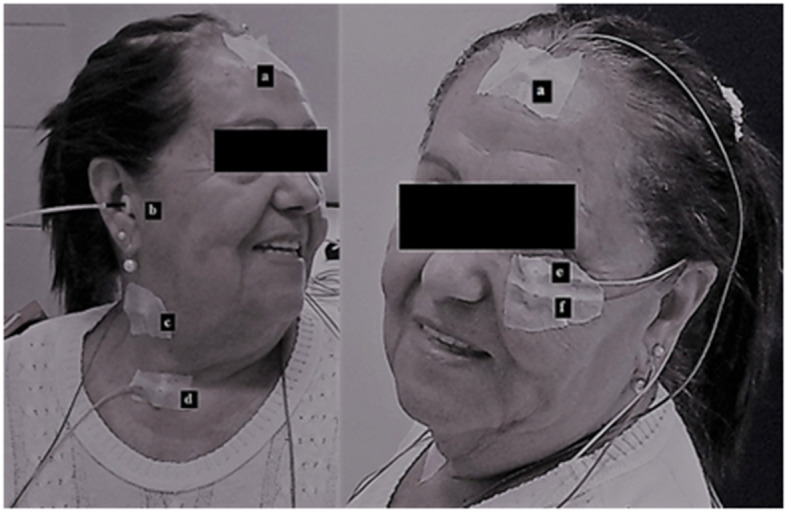
Simultaneous cervical and ocular VEMP: **(a)** ground electrode, **(b)** auditory stimulus, **(c)** active electrode on channel 2 at the anterior border of the sternocleidomastoid muscle in its upper third, **(d)** reference electrode on channel 2 at the sternal notch region, **(e)** active electrode on channel 1 below the lower eyelid, and **(f)** reference electrode on channel 1 below the active electrode.

For oVEMP recordings, the active electrode (model Grass Gold Electrodes Silicone, Natus) on channel 1 was placed ~1 centimeter (cm) below the lower eyelid, and the reference electrode was placed distant approximately 1 cm from the active electrode. The ground electrode was placed on the forehead (Fpz). For cVEMP recording, the active electrode on channel 2 was placed on the opposite side of channel 1, on the anterior border of the sternocleidomastoid muscle in its upper third, while the reference electrode was placed in the sternal notch region (Figure 1).

The participants were instructed to sit on the chair and keep their heads rotated to the opposite side of the stimulated ear in order to contract the sternocleidomastoid muscle. We compared reflexes of approximately similar size, where the cVEMP asymmetry between sides was <34%. At the same time, the participant was instructed to look at a stationary target located on the wall in front of him/her and then immediately at a fixed point located above the target, which formed a vertical viewing angle of approximately 30° above the horizontal plane. The oVEMP and cVEMP protocols are available at dx.doi.org/10.17504/protocols.io.zmzf476.

**Figure 2:**
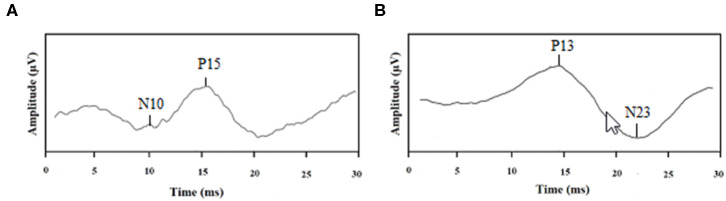
Examples of tracings: **(A)** normal ocular VEMP and **(B)** normal cervical VEMP.

The oVEMP trace is a biphasic wave. The two phases are characterized by a negative peak with an average latency of 10 milliseconds (ms) (N10), followed by a positive peak with an average latency of 15 ms (P15), which is known as N10–P15. The cVEMP trace consists also of a wave with two phases. The first peak is positive with an average latency of 13 ms (P13), followed by a negative peak with an average latency of 23 ms (N23), which it known as P13–N23 (Figure 2).

### Soleus VEMP (sVEMP)

Galvanic stimulation has been considered a tool to activate the vestibular system inducing both ocular and postural movements (21). The stimulus usually varies from 2 to 4 mA and the duration goes from 20 to 400 ms. The higher the current, the shorter the time of the stimulus (21, 22, 28). The EMG response, that is the VEMP, can be recorded in a muscle involved in either the ocular or the postural movements. The EMG response in the soleus muscle is characterized by a short latency (SL) wave, beginning at around 60 ms, followed by a response in the opposite direction at medium latency (ML), beginning at around 100 ms (21, 22, 29); Both SL and ML responses can only be detected if the muscle is actively contracting (21). In the soleus, SL and ML responses to the transmastoid stimulation are clearest when the subjects head is turned to one side. Both SL and ML responses invert when the head is turned to the opposite side or when the cathode and anode are reversed (21, 22). Cathodal stimulation has been shown to excite, and anodal stimulation to inhibit the vestibular nerve afferent discharge (30). The responses that invert in response to stimulation of opposite polarities and have latencies similar to those previously described, are taken to be of vestibular origin (21, 22).

In the present study, the galvanic vestibular stimulation (GVS) was characterized as a direct, monophasic, and rectangular current with an intensity of 2 mA and duration of 400 ms (model EvP4/ATCPlus, Contronic, Ltd., Pelotas, Brazil). The galvanic stimulus was offered at randomized intervals of 4–5 s and responses to 120 stimulations were measured. The bipolar current was applied on the mastoid processes using self-adhesive, circular surface electrodes (3 cm diameter; model CF3200, Valutrode, Axelgaard, Fallbrook, CA).

For transmastoid stimulation, the two current polarity settings were cathode left and anode right (CLAR) and cathode right and anode left (CRAL). The stimulation polarity was controlled by a computer. For each test, four sets of 30 stimuli were applied and distributed, resulting in 30 responses recorded from the left lower limb (15 CLAR and 15 CRAL stimuli) and 30 responses recorded from the right lower limb (15 CLAR and 15 CRAL stimuli). This procedure was repeated for each leg to ensure data replication.

During the acquisitions, the subjects stood barefoot on a flat surface with their eyes closed, feet close together, and bodies leaning forward to contract the soleus muscle. To induce a stronger response, subjects were instructed to turn their heads approximately 90° to the side contralateral to the leg undergoing EMG response recording.

**Figure 3:**
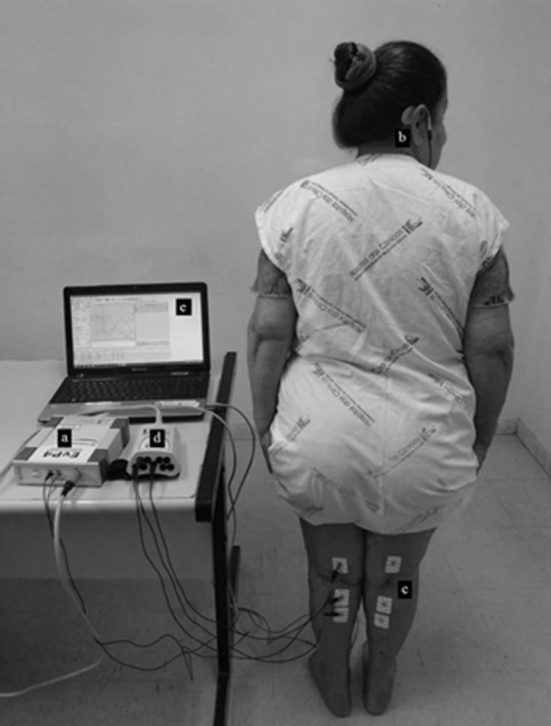
Vestibular-evoked myogenic potential triggered by galvanic vestibular stimulation. The figure shows: the standing position of the patient (barefoot on a hard flat surface with eyes closed, feet close together, and body leaning forward in order to cause the soleus muscle contraction); the equipment used for stimulus generation **(a)**; the electrode positions for GVS **(b)**; the electrode position for electromyography on the soleus muscle **(c)**; the equipment for signal processing **(d)**; and the laptop **(e)** connected to **(a)** and **(d)**.

The EMG response triggered by GVS was measured using self-adhesive electrodes (model 2223BRQ, 3M, Saint Paul, MN). A pair of recording electrodes were placed bilaterally 5 cm below the popliteal fossa, which coincides with the position over the soleus muscles. Each pair was placed vertically distant 5 cm from each other. This distance can vary from 3 to 10 cm, according to the best recorded wave (21, 22). A reference electrode was placed on the back of the thigh at approximately 10 cm above the upper most recording electrode. The sVEMP was first measured in the left leg and then in the right leg. The tests were performed with a 2-min resting interval to avoid muscle fatigue (Figure 3).

The EMG signals were measured, corrected, with a bandpass filter of 10 to 1,000 Hz, and digitized at a sampling frequency of 5,000 Hz. Data were recorded during 500 ms, starting 100 ms before GVS. The EMG responses to 15 consecutive stimuli associated with each polarity configuration (i.e., CLAR and CRAL) were averaged to produce the final traces. The sVEMP protocol is available at dx.doi.org/10.17504/protocols.io.nxbdfin.

**Figure 4:**
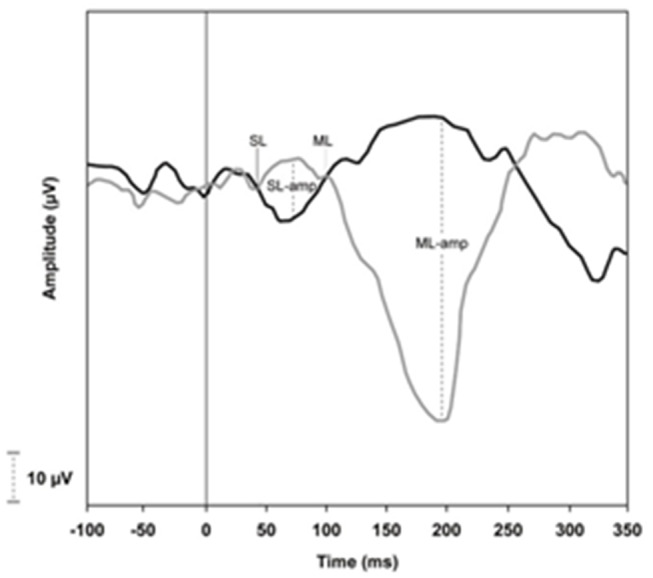
Example of traces obtained by VEMP recording from soleus muscle.

The EMG tracings were analyzed for the time of onset, in milliseconds, of the short latency response (SL), and the mean latency response (ML). Following the superimposition of traces with inverted polarity (i.e., CRAL and CLAR), the point where the traces diverged from the EMG baseline, which marked the onsets of SL and ML, could be visualized and measured by a cursor. The first trace divergence, which occurred at approximately 50 ms, marked the onset of the SL response. Following this, the traces returned to baseline and then diverged again. The second trace divergence, which occurred at approximately 100 ms, marked the onset of the ML response. The end of this response was defined as the point at which traces return to the baseline. To obtain a single value of the components of SL and ML, it was considered the worst response between the right and left sides (Figure 4).

### Statistical Analysis of Data

The parameters considered in the VEMP analysis are the latency and amplitude of the waves. However, the amplitude can vary according to age, muscular strength (31), and cochlear diseases (32, 33). Although these variables were controlled in the present study, muscular atrophy in the lower limbs is characteristic of HAM and could therefore act as a possible bias, inducing false positive results. Therefore, the amplitude was not a variable in the analysis.

VEMP results were classified as normal and altered. The results with latency prolongation or no response were considered as altered. The VEMP latency prolongation was defined as a delay of 2.5 standard deviation (SD) when compared to the values of a normal control population, according to the American Society of Encephalography and Evoked Potentials' criteria for evoked potentials (34). The latency values used in this study for the purpose of comparison were the VEMP latencies of the HTLV-1-seronegative group. The validation of the analyzed reference values was guaranteed by comparing these latencies with parameters already established in other national and international peer reviews (16, 35). Among the tested participants, in order to obtain only one value for the peaks N10-P15 (oVEMP), P13-N23 (cVEMP), and SL-ML (sVEMP), the worst response between the right and left sides was considered. Statistical analysis was performed using the Statistical Package for Social Sciences (SPSS), version 20.0. The normality of the samples was assessed using the Kolmogorov-Smirnov and Shapiro-Wilk tests. The results of oVEMP, cVEMP, and sVEMP were compared between the groups infected and not infected by HTLV-1. The comparison between groups was performed using the Kruskal-Wallis test, ANOVA test, Chi-square or Fisher's Exact test, Kruskal-wallis with Bonferroni correction, and ANOVA with Bonferroni correction. The adopted level of significance was 5% (*p* ≤ 0.05).

The receiver operating characteristic curve (ROC) was performed with the objective of identifying the sensitivity and specificity for each latency cutoff of oVEMP, cVEMP, and sVEMP, considering the neurological examination as the gold standard.

## Results

The general characteristics of the studied sample can be seen in Table 1. The groups were similar in relation to gender (*p* = 0.549) and age (*p* = 0.069).

**Table 1:** General characteristics of the patients with HTLV-1 associated myelopathy (HAM), HTLV-1-asymptomatic carriers and seronegative controls, EDSS and OMDS disability scales (*n* = 90).

**Variable**	**G1 (*n* = 30)**	**G2 (*n* = 30)**	**G3 (*n* = 30)**	***p* value**
Age	53 [50/55]	56.5 [49/60]	57 [52/59]	0.069^a^
EDSS	0 [0/0]	0 [0/0]	1.75 [1.5/4.5]	<0.001^a^
OMDS	0 [0/0]	0 [0/0]	1.0 [1.0/3.0]	<0.001^a^
**Gender**				
Female	20 (66.7)	18 (60.0)	22 (73.3)	
Male	10 (33.3)	12 (40.0)	8 (26.7)	

*G1, HTLV-1 seronegative; G2, HTLV-1-asymptomatic carriers; G3, HAM; n, number of participants; median [1° quartile / 3° quartile] for continuous variables with asymmetric distribution; absolute number (percentage) for categorical variables; EDSS, extended functional disability scale; OMDS, Osame motor disability scale*.

a *Kruskal-Wallis Test (p ≤ 0.05)*.

b *Chi-square Test (p ≤ 0.05)*.

The comparison of oVEMP, cVEMP, and sVEMP among the groups regarding the delay of latencies of each peak of the biphasic waves is shown in Table 2. The statistical analysis has showed that, in cVEMP and sVEMP, the change has started in the first component of the wave, followed by a delay in the second component. This can be seen by comparing the latencies between HTLV-1-asymptomatic group and the controls. The latency of the first components were different (P13, *p* = 0.039; SL, *p* < 0.001), while the latency of the second components have remained similar between groups (N23, *p* = 0.575; ML, *p* = 0.187). The comparison between HTLV-1-asymptomatics and HAM has showed a delay in the first components for both groups (P13, *p* = 1,000; SL, *p* = 0.199), and the second components have not changed comparing to the controls. With regard to oVEMP, the prolonged latency was observed only in the HAM group.

**Table 2:** Comparison among the groups HTLV-1 associated myelopathy (HAM), HTLV-1-asymptomatic carriers, and seronegative controls regarding the VEMP latency recorded in ocular, cervical, and soleus muscles (*n* = 90).

EMG waves latencies^*^	**G1 (*n* = 30)**	**G2 (*n* = 30)**	**G3 (*n* = 30)**	***p* value**	**Comparison groups**	*p* value^**^
**Ocular VEMP^a^**						
N10	10.63 [9.96/11.00]	10.38 [9.91/10.93]	10.40 [9.95/13.60]	0.675^d^	–	^−^
P15	15.39 (0.63)	16.11 (2.08)	18.47 (3.03)	<0.001^e^	G1 X G2	0.461
					G1 X G3	<0.001
					G2 X G3	0.001
**Cervical VEMP^b^**						
P13	12.93 (0.92)	13.71 (0.97)	13.99 (1.73)	0.006^e^	G1 X G2	0.039
					G1 X G3	0.013
					G2 X G3	1.000
N23	22.28 (1.32)	23.27 (2.77)	26.59 (6.41)	0.005^e^	G1 X G2	0.575
					G1 X G3	0.004
					G2 X G3	0.043
**Soleus VEMP^c^**						
SL	55.81 (3.47)	59.62 (4.25)	62.25 (3.10)	<0.001^e^	G1 X G2	<0.001
					G1 X G3	<0.001
					G2 X G3	0.199
ML	111.88 (7.36)	115.87 (9.13)	128.90 (3.48)	<0.001^e^	G1 X G2	0.187
					G1 X G3	<0.001
					G2 X G3	<0.001

*G1, HTLV-1 seronegative; G2, HTLV-1-asymptomatic carriers; G3, HAM*.

* *Components of the electromyographic (EMG) wave: N10 and P15 for ocular VEMP; P13 and N23 for cervical VEMP; SL (short latency); ML (medium latency) for soleus VEMP; n = number of participants; median [1° quartile / 3° quartile] for continuous variables with asymmetric distribution; mean (standard deviation) for continuous variables with symmetric distributions*.

a *The cases of lack of latency were excluded from this analysis: 15 HAM in ocular VEMP*;

b 25 HAM and 9 HTLV-1-asymptomatic carriers in cervical VEMP, and

c *20 HAM and 6 HTLV-1-asymptomatic carriers in soleus VEMP*.

d *Kruskal-Wallis Test (p ≤ 0.05)*.

**e *Anova (p ≤ 0.05)*.

**Figure 5:**
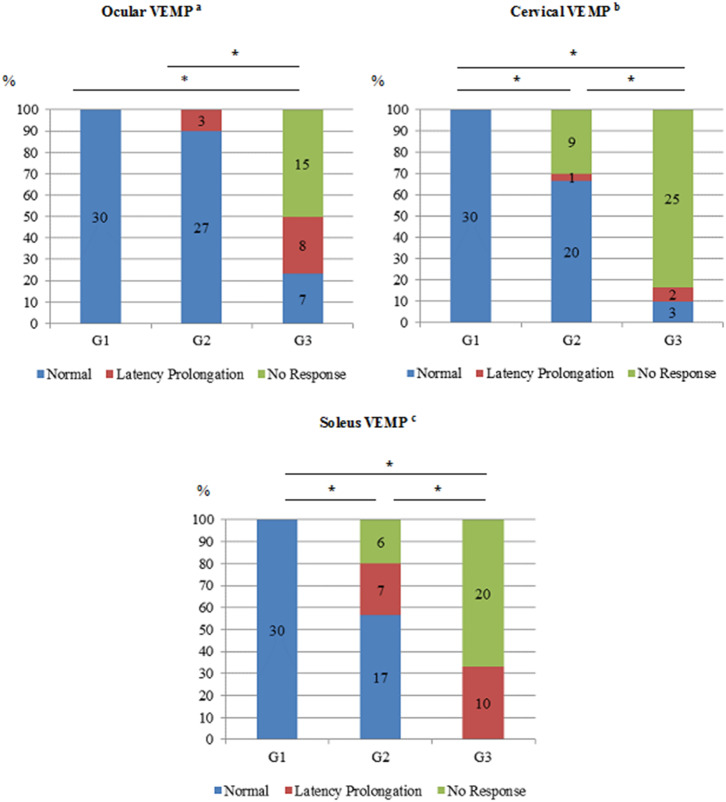
Comparison of VEMP recorded from ocular, cervical, and soleus muscles in the groups HTLV-1 seronegative (*n* = 30), HTLV-1-asymptomatic carriers (*n* = 30) and HAM patients (*n* = 30). G1, HTLV-1 seronegative; G2, HTLV-1-asymptomatic carriers; G3, HAM; The altered responses were categorized as the latency prolongation and the lack of latency of N10-P15 for ocular VEMP^a^; P13-N23 for cervical VEMP^b^; SL-ML for soleus VEMP^c^. ^*^p ≤ 0.001, Fisher's Chi-Square or Exact Test.

Figure 5 shows the comparative analysis of oVEMP, cVEMP, and sVEMP, considering the latencies categorized as normal, latency prolongation, and no response. It shows the progressive VEMP alteration from the asymptomatic stage to HAM and from the lumbar spinal damage, detected by sVEMP, to cervical damage, detected by cVEMP, and a more frequent mesencephalic alteration, detected by oVEMP, in patients with HAM as compared to HTLV-1-asymptomatic carriers.

In Table 3, oVEMP, cVEMP, and sVEMP were categorized as normal and altered and the results are presented as an analysis between-groups, according to VEMP stratification of the altered results per group. When a concomitant alteration in VEMP recorded from the three muscles was considered, HTLV-1-asymptomatic group did not differ from the normal controls (*p* = 0.983), but it was different from the HAM group (*p* = 0.001).

**Table 3:** Stratified between-groups comparison of VEMP recorded in ocular, cervical, and soleus muscles of HAM (*n* = 30), HTLV-1-asymptomatic carriers (*n* = 30), and HTLV-1-seronegative (*n* = 30) groups.

**Electrophysiological Evaluation (VEMP)**	**G1 (*n* = 30)**	**G2 (*n* = 30)**	**G3 (*n* = 30)**	*p* value^*^	**Comparison groups**	*p* value^**^
	***N*** **(%)**	***N*** **(%)**	***N*** **(%)**			
Normal	30 (100.0)	12 (40.0)	0 (0.0)	<0.001	G1 X G2	0.002
					G1 X G3	<0.001
					G2 X G3	0.003
Only oVEMP altered	0 (0.0)	0 (0.0)	0 (0.0)	–	–	–
Only cVEMP altered	0 (0)	5 (16.7)	0 (0.0)	0.925	–	–
Only sVEMP altered	0 (0)	7 (23.3)	0 (0.0)	0.876	–	–
oVEMP + cVEMP altered	0 (0)	0 (0.0)	0 (0.0)	–	–	–
oVEMP + sVEMP altered	0(0)	1 (3.3)	3 (10.0)	0.741	–	–
cVEMP + sVEMP altered	0(0)	3 (10.0)	7 (23.3)	0.689	–	–
oVEMP + cVEMP + sVEMP altered	0(0)	2 (6.7)	20 (66.7)	0.001	G1 X G2	0.983
					G1 X G3	0.004
					G2 X G3	0.001

*G1, HTLV-1 seronegative; G2, HTLV-1-asymptomatic carriers; G3, HAM; oVEMP, ocular VEMP; cVEMP, cervical VEMP; sVEMP, soleus VEMP*.

* Fisher's Chi-Square or Exact Test (p ≤ 0.05)/

** *Bonferroni Test*.

**Figure 6:**
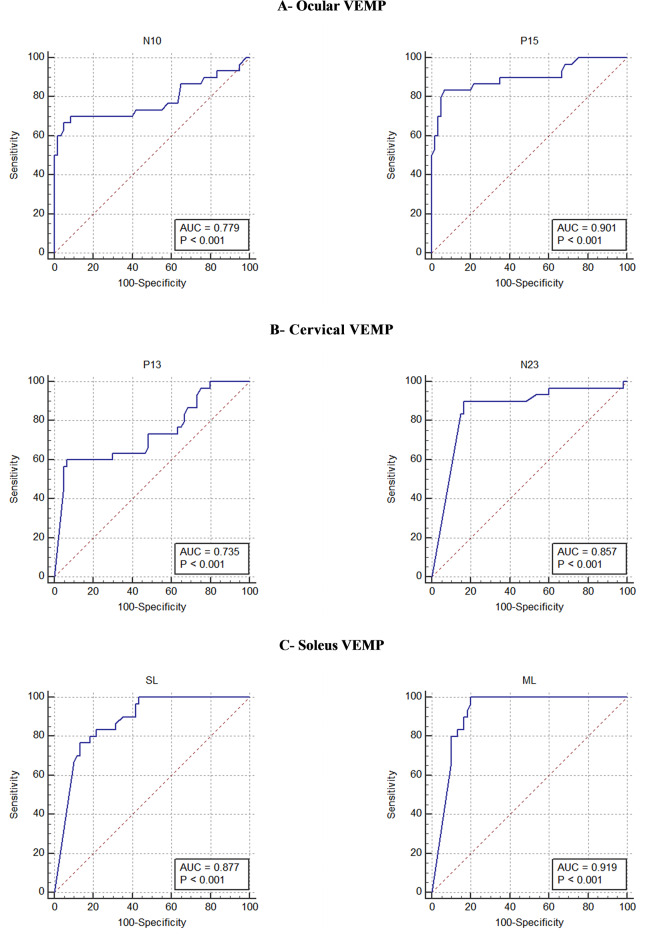
The ROC curve of the components of the waves N10-P15 of ocular VEMP, P13-N23 of cervical VEMP, and SL-ML of soleus VEMP, considering neurological examination as the gold standard. AUC, area under the curve.

To evaluate the use of VEMP tests in clinical practice, VEMP latencies were then compared to the neurological examination as the gold standard. We have constructed ROC curves to evaluate latency prolongation of N10-P15 for oVEMP, P13-N23 for cVEMP, and SL-ML for sVEMP (Figure 6). The better cut-off points regarding the HTLV-1 infected population were 11 ms for N10, with a sensitivity of 70.0% and a specificity of 91.7%; 16 ms for P15, with a sensitivity of 83.3% and a specificity of 93.3%; 15 ms for P13, with a sensitivity of 60.0% and a specificity of 93.3%; 25 ms for N23, with a sensitivity of 90.0% and a specificity of 83.3%; 65 ms for SL, with a sensitivity of 76.7% and specificity of 86.7%; and 123 ms for ML, with a sensitivity of 100.0% and a specificity of 80.0%. The criterium of using the worst EMG response between the sides contributed to increase the sensitivity of the test, since only one altered side was enough to categorized the patient as altered whereas the normal result was categorized like that only when the waves were truly normal in both sides.

Figure 6 shows that the area under the ROC curve was greater for the second component of the biphasic waves in VEMP of the three tested muscles. Therefore, this component was shown to be the most reliable to define early EMG changes.

## Discussion

The HAM diagnosis is based on a set of clinical criteria established by an international consortium in 2006 (24). However, the clinical manifestations related to HAM seem to precede the diagnosis of definite HAM in years, in such a way that they are more frequent in the considered asymptomatic HTLV-1-carriers than in the non-infected individuals (36–38).

More recently, a longitudinal study based on eight years of follow-up has confirmed that the asymptomatic carrier has presented elevated morbidity related to HTLV-1, such as, autonomic changes, including alteration in bowel habits, urinary incontinence or urgency, erectile dysfunction, as well as motor disabilities (39). Thus, the diagnosis of HAM based on clinical criteria establishes the final event of neurological sequelae as the initial mark to define the diagnosis of a disease of chronic evolution. In this context of a late diagnosis for HAM, VEMP is a very useful electrophysiological tool, as it contributes to the detection of alterations related to HTLV-1 before a visible alteration in the neurological examination. VEMP tests the vestibulo-ocular reflex that is related to the stabilization of the image in the retina with the movement of the head and tests the vestibulospinal and reticulospinal tracts related to the postural control (40).

Regarding oVEMP, it is assumed that the neural connections involved in EMG response are mesencephalic (18, 19, 26, 41). The altered responses, such as the latency prolongation or the absence of EMG waves, depend on the disorganization of the primary afferents involved in the vestibulo-ocular reflex (18, 19). In this study, we have found that oVEMP was more frequently altered in the HAM group when compared to the HTLV-1-asymptomatic group (Figure 5). In addition, the worst changes were seen in the HAM group. While in this last group the lack of EMG response was the most common change, in the asymptomatic group, this alteration was not found. The oVEMP response in the asymptomatic group did not differ from the controls. These results reinforce the hypothesis that midbrain is compromised in HAM but not in HTLV-1-asymptomatic carriers (7, 12–14).

Regarding cVEMP, as expected, EMG responses were worse in the HAM group when compared to the HTLV-1-asymptomatic group. These data confirm that HAM compromises the cervical spine, although the alterations have been worse in the thoracolumbar region (42, 43). The change from a latency prolongation to a lack of EMG waves suggests that an increase in the neuronal damage occurred (16, 22, 32). This premise can be confirmed by the analysis of the frequency of absent EMG response in the HAM group comparing to the asymptomatic group.

The analysis of sVEMP shows that, in HAM group, all the participants have had altered responses, with a higher frequency of absence of EMG waves (Figure 5). In the HTLV-1-asymptomatic group, the comparison of cVEMP and sVEMP results (Table 2 and Figure 5) shows that the electrophysiological alterations were already present in a significant proportion of participants in both cervical and lumbar levels, although the frequency of changes was much higher in HAM group. In fact, VEMP recorded from different muscles may be used to clarify the range of the neurological injury (45).

In sVEMP, the first component of the wave (SL) is assumed to result from a synchronous discharge of a common supraspinal structure, which means, the reticulospinal and the vestibulospinal tract, while the second component (ML) represents the polysynaptic synchrony (21, 22). In accordance with the present study, previous studies have already shown that ML is the best component to discriminate changes since this peak is easier to define with the best intrarater and interrater agreement and presents the best area under the ROC curve comparing to SL (47–49). Conversely, SL can be often indistinguishable from the baseline and its measurement has presented the worst interrater correlation comparing to ML (45, 47–49).

The Table 3 shows a higher frequency of simultaneous changes in oVEMP, cVEMP, and sVEMP in HAM group. This fact has confirmed the greater spinal impairment in HAM when compared to the group with asymptomatic infection. The changes in oVEMP have occurred only in HAM group, which indicates a midbrain involvement. This finding is precisely in accordance with the best knowledge about HAM physiopathology and reinforces the validity and accuracy of VEMP for clinical use (44–49). In this study, the best contribution of VEMP in the evaluation of the HTLV-1 population was to the asymptomatic infection. A subclinical diagnosis of neurological impairment seems to be possible using VEMP, and it will make difference when the scientific progress comes to a more effective treatment of HAM. Our results allow to infer about a pattern of VEMP changes that has occurred. The deficit has started with subtle latency delay and has progressed through degradation of the response and has ended with an absent response. Considering cVEMP and sVEMP, the EMG alteration has started at the first component (P13, and SL), followed by a latency prolongation at the second component (N23, and ML) until the EMG response has become absent.

In short, we have found that the neurological damage related to HTLV-1 follows an ascending progression since the subclinical stage. The image of the spinal atrophy in the advanced HAM confirms the ascendant damage as it shows that the spinal cord is thinner at the thoracolumbar region than at the cervical one (44). Considering the length of the central pathway, sVEMP has represented a better tool for the early diagnosis of HAM than oVEMP and cVEMP, since these lasts show functional degradation in structures that are anatomically higher in CNS and sVEMP shows degradation that is located in a lower level. On the other hand, oVEMP was useful for the early detection of midbrain changes found in HAM (13, 14).

Vestibular-evoked muscle responses have been used to evaluate the spinal cord in trauma and other neuroinfectious diseases (45, 48). The Schistosomal myeloradiculopathy (SMR) is the most severe and disabling ectopic form of *Schistosoma mansoni* infection and represents 6% of non-traumatic transverse myelopathies in endemic areas (50). The sVEMP triggered by galvanic stimulation was shown to be a promising tool to add electrophysiological information about the spine of patients with chronic SMR. The sVEMP was a reliable and reproducible method to define the integrity of the vestibulospinal tract, with an excellent intrarater and interrater agreement and reliability. Both in HAM and SMR, the component ML was shown to be the most reliable to define alteration (47, 48).

One limitation of the present study was the lack of control of the height and the gender as potential confounding variables. Women have predominated in this study and the ML is more prolonged in women than in men (19). Moreover, VEMP latency has been found to correlate with height (33). Therefore, the cutoff values for the latencies considered in the present study deserve caution regarding the use of these values for the validation of VEMP in different groups of people under different conditions. To avoid this problem, in case of using VEMP in clinical practice, it is desirable to conduct studies that assess VEMP latencies in local healthy people under local conditions for the definition of reliable cutoffs (29).

Another limitation was its transversal design. Although the GIPH cohort includes incident cases of HAM, the participants of the present sectional analysis were not submitted to the entire battery of VEMP tests when they were asymptomatic carriers and afterwards evolved to HAM. Therefore, we cannot make any supposition, based on the present data, about the prognostic value of VEMP alterations within HAM development. The absence of a battery of neurocognitive tests, in addition to the clinical examination, was also a limitation. We have constructed the ROC curve based on the neurological examination, but it would be interesting to analyze the correlation of HTLV-1 cognitive alterations and oVEMP. We do not know if the HTLV-1 infected population with a midbrain alteration would also present a greater risk for cortical alterations. This question deserves a properly designed study to remedy this matter.

## Conclusion

VEMP analysis of different muscles showed that in HAM the neurological damage has occurred in the spine as well as at the midbrain level. In the asymptomatic carriers, a sub-clinical damage has followed an ascending progression, since changes in VEMP were more frequent in the lumbar as compared to the cervical spine. Thus, VEMP recorded from the soleus muscle, compared to the cervical and ocular muscles, was a better clinical tool for the early diagnosis of neurological changes in a HTLV-1-infected population.

## Data Availability Statement

All datasets generated for this study are included in the article/Supplementary Material.

## Ethics Statement

The studies involving human participants were reviewed and approved by This research was conducted in accordance with the principles expressed in the Declaration of Helsinki and was approved by the Research Ethics Committee from Universidade Federal de Minas Gerais (COEP UFMG), logged under protocol number CAAE 92928518.3.0000.5149. The patients/participants provided their written informed consent to participate in this study. Written informed consent was obtained from the individual(s) for the publication of any potentially identifiable images or data included in this article.

## Author Contributions

TS: conceptualization, data curation, formal analysis, funding acquisition, methodology, project administration, resources, validation, visualization, writing-original draft, writing-review and editing. MR and LM: conceptualization, formal analysis, funding acquisition, methodology, project administration, resources, supervision, validation, visualization, writing-review and editing. LL and JC: validation, visualization, writing-review and editing. RS: investigation and validation. DU: data curation, formal analysis, funding acquisition, methodology, project administration, resources, supervision, validation, visualization, writing-original draft, writing-review and editing.

## Conflict of Interest

The authors declare that the research was conducted in the absence of any commercial or financial relationships that could be construed as a potential conflict of interest.
